# Employment preferences of obstetricians and gynecologists to work in the district hospitals: evidence from a discrete choice experiment in Nepal

**DOI:** 10.1186/s12960-019-0427-8

**Published:** 2019-12-09

**Authors:** Bishnu Gautam, Vishnu Prasad Sapkota, Rajendra Raj Wagle

**Affiliations:** 1 Central Department of Public Health (CDPH), Institute of Medicine, Tribhuvan University, Kathmandu, Nepal; 2 Department of Obstetrics and Gynaecology, Lumbini Zonal Hospital, Rupandehi, Butwal, Nepal; 3 Department of Community Medicine, Maharajgunj Medical Campus, Tribhuvan University, Kathmandu, Nepal

**Keywords:** Career choice, Discrete choice experiment, Job preference, Nepal, Obstetrician and gynecologist, Rural health service

## Abstract

**Background:**

A mismatch between the requirement and annual production of obstetricians and gynecologists (OBs-GYNs) was observed in Nepal. On top of that, recruitment and retention of OBs-GYNs is a pressing problem, especially in district hospitals of Nepal. In this connection, evidence on the job priorities and preferences of OBs-GYNs, which is currently lacking in Nepal, would help in policymakers in devising recruitment and retention strategies in these hospitals. This study, therefore, aims at exploring the most relevant job attributes that OBs-GYNs would prefer to work in the district hospitals of Nepal using a discrete choice experiment (DCE) technique.

**Methods:**

Job attributes relevant to design the questionnaire were identified using keyinformant interviews and focusgroup discussions with policymakers and top managers. Then, 48 choice sets were developed using a fractional factorial design. Using these unlabeled choice sets, a DCE was conducted among 189 OBs-GYNs. The multinomial logistic regression model was used to estimate the marginal utilities and other model parameters. The willingness to pay/accept estimates was also measured for each job attribute.

**Results:**

OBs-GYNs preferred the presence of a full team at the workplace (OB-GYN, pediatrician, and anesthesiologist), provision of primary and secondary education for children, and opportunity of private practice. On the other hand, a few job attributes such as a higher duration of service in district hospitalsand the provisions of a car allowance were preferred less by the respondents. Results from the marginal utility by the OBs-GYNs would be open to trade among the attributes.

**Conclusions:**

The job attributes identified as incentives in this study should be included in a package to attract OBs-GYNs to serve in district hospitals of Nepal rather than offering a standard incentive package to all health workers. Similarly, this study confirmed the importance of the combination of non-monetary and monetary interventions in attracting and retaining health workers in district hospitals of Nepal.

## Background

Despite a huge achievement in reducing maternal mortality over a decade, a disproportionately poorer maternal health indicator is observed in many districts of Nepal where Comprehensive Emergency Obstetrics and Neonatal Care (CEONC) services are poorly organized [1]. Therefore, to make further progress in this area, better access to well-equipped hospitals and the availability of obstetricians and gynecologists (OBs-GYNs) can be pivotal to reducing maternal deaths and improving maternal health indicators [2]. However, the proportion of sanctioned positions of OBs-GYNs that are fulfilled remains unclear. A national survey of health workers in 2011 identified that 7% of sanctioned positions were vacant for all kinds of health workers [3]. The same study reported that the percentage of vacant positions was 29% for medical officers. This statistic reflects the challenges in recruitment and retention of medical doctors in Nepal, especially in the health facilities of the district hospital and below. The fulfillment of the sanctioned positions of OBs-GYN is also poor, if not worse. A similar pattern of mismatch in the requirement and supply of doctors, including obstetricians, is also observed in low- and middle-income countries particularly in rural areas [4, 5]. The reason for this includes a complex interplay between several variables at the individual, local, and work environment and national and international level of care [6]. However, the factors that determine the doctors’ unwillingness to serve at a district hospital vary between countries.

Currently, Nepal is in the mission of increasing the recruitment and retention of OBs-GYNs in district hospitals to speedup the achievements in the maternal and child health indicators. For this purpose, district hospitals—an operational-level decision-making unit (DMU) for the health services of its coverage population and located in district headquarters—are first referral and primary service delivery institutions. The district headquarter also serves as the market center for the district population. Most of the district headquarters are located in semi-urban areas with transportation linkages and availability of basic amenities. However, some of the districts as such are categorized as low-Human Development Index (HDI) districts. The Government of Nepal introduced the CEONC program in 56 districts [7]. Under this program, the Family Health Division provides a fund to district hospitals to cover high payment to doctors and procure and maintain equipment and drugs in addition to the other interventions. The presence of OBs-GYNs in district hospitals is pivotal to the implementation of the CEONC program. The OBs-GYNs cover those who have completed the Master’s Degree in Obstetrics and Gynecology (MD), Diploma in Obstetrics and Gynecology (DGO), or Fellowship in Royal College of Obstetrics and Gynecology (FRCOG). The OB-GYN will perform the surgical procedure, inpatient and outpatient department management of obstetrics and gynecological-related diseases, andsurgical procedures likelower segment cesarean section (LSCS) and laparotomy for ectopic pregnancy or any other emergency condition in their job setting.

However, the problem is to identify what incentives are the most effective in encouraging doctors to serve in those district hospitals in order to make efficient utilization of such funds. In the literature, financial, educational, and professional incentives have been reported to attract doctors in those institutions [8, 9], but which combination of these financial and non-financial incentives is the most effective is a crucial question. Identification of the most effective job package with a series of trial and error can both be costly and ineffective. In this setting, the discrete choice experiment (DCE) is an advantageous approach. The approach was introduced into health economics in the early 1990s, and it should be considered by policymakers and adopted as a useful tool while evaluating health care intervention [10]. The result provides evidence that the DCE approach can value increase satisfaction [11]. Under DCE, participants are provided with competing sets of choices to choose from, and based on this, valuation of each choice attribute is computed and expressed in terms of willingness to pay/accept [12, 13].

Against this background, the study aims at estimating the relative weight OBs-GYNs place on different job attributes while selecting placement in two or more hypothetical jobs in an ideal district hospital of Nepal using the DCE technique.

## Methods

### Study setting

This study was carried out among the OBs-GYNs serving in Nepal during the period of 2015/2016 covering all the OBs-GYNs, and therefore, it was a census of OBs-GYNs. In Nepal, the public health system includes central tertiary hospitals, zonal and district hospitals, Primary Health Care Centre (PHCC), and Health Post (HP). Nepal has seven provinces and 77 districts, and each district has district hospitals, PHCCs, and HPs. The district hospital is the first referral center from the PHCC and HP. Within this organogram, the Government of Nepal has implemented the CEONC program in 69 districts out of which only 61 are running satisfactorily. In district hospitals, there is a provision of OB-GYN, anesthesia assistant, and staff nurse to successfully run the CEONC program. The hospitals that are reported not performing well are located in low-Human Development Index (HDI) districts in the hilly and mountainous terrain of Nepal.In addition to this, OBs-GYNs also serve in private hospitals and academia. OBs-GYNs were included from all these settings for this study. This study was carried out among all the OBs-GYNs serving in Nepal during the period of 2015/2016, and therefore, it was a census of OBs-GYNs.

### Measures

The questionnaire for this survey was developed in multiple stages. In the first stage, a series of key informantinterviews (KIIs) and focus group discussions (FGDs) were conducted following literature reviews to identify the conceptual attributes relevant to OBs-GYNs’ job preferences. Therefore, FGDs and KIIs aimed to find the policy-relevant job attributes that are useful to the policymakers and also relevant to the respondents. Three FGDs were conducted with OBs-GYNs from tertiary maternity care hospitals located in different parts of the country: Bharatpur Hospital, Chitwan; Lumbini Zonal Hospital, Rupandehi; and Tribhuvan University Teaching Hospital, Kathmandu. Similarly, we also carried out KIIs with four OBs-GYNs from Paropakar Maternity Hospital—a tertiary referral center for maternity care. FGDs and KIIs aimed to find the policy-relevant job attributes that are useful to policymakers and relevant to the respondents. Information from FGDs and KIIs were grouped in two domains, i.e., individual motivation (family welfare, salary, accommodation, education of children, security, and facility in the hospital) and institutional features (teamwork, capacity building, logistics, referral mechanism, multidisciplinary team, and technology). From these interviews and discussions, eight attributes and corresponding levels were identified for the questionnaire (shown in Table 1).

**Table 1:** Attributes and levels used in the discrete choice experiment

Attribute	Levels
Minimum duration of service	2 years
	3 years
	4 years
	5 years
Provision of education for children	No educational provision
	Provision of primary education
	Provision of secondary education
Opportunities of professional development	Not available
	Available
Availability of technological support	No technological support available
	Either telemedicine or video conferencing support available
	Both telemedicine and video conferencing support available
Team in the job setting	Team of OB-GYN, pediatrician, and anesthesiologist
	Team of OB-GYN and anesthetic assistant
Monthly salary	NPR 100 000 ($1 000)
	NPR 125 000 ($1 250)
	NPR 150 000 ($1 500)
	NPR 175 000 ($1 750)
	NPR 200 000 ($2 000)
	NPR 225 000 ($2 250)
Opportunity for private practice	Private practice not allowed
	Private practice allowed
Provision of car allowance	Car allowance not provided
	Car allowance provided

The second phase of questionnaire development was the generation of choice sets. Combining attributes and attribute levels showed that 3456 alternatives were found possible using full factorial design. Such a large number of alternatives were not practical, and many of the alternatives were not relevant. Therefore, this study used a fractional factorial design to reduce the number of choice sets to a manageable level. The rotation method uses the orthogonal main-effect array as the first alternative in each choice set; this method creates one or more additional alternatives by adding a constant to each attribute level of the first alternative; the *k*th (≥ 2) alternative in the *j*th (= 1, 2, … , *J*) choice set is created by adding one to each of the *m* attributes in the (*k*−1)th alternative in the *j*th choice set. If the level of the attribute in the (k −1)th alternative is maximum, then the level of the attribute in the *k*th alternative is assigned the minimum value. These choice sets were reviewed for relevance before translating them into the questionnaire. We implemented this process using *support. CE* package in R [14]. The key requirements of fractional factorial design: the orthogonality (attribute levels are independent of each other), level balance (attribute levels appear with the same frequency), and minimal overlap (attribute levels do not repeat across alternatives) were maintained. Finally, 48 choice sets were developed which were divided into six blocks; each block contains eight choice sets. This means that each respondent got eight choice sets to respond. We used a generic design DCE, in which the job postings were not labeled as rural or urban and respondents were requested to assume that they were looking for a new job and asked to choose between two advertised job postings in government district hospitals. The unlabeled choice set design (i.e.,“job A,”“job B,” and “none of the above”) was used (a sample of choice set is available in Table2). The questionnaire was pretested in a sample of 30 OBs-GYNs, including those who participated in the FGDs and KIIs. The data collected from pretesting were analyzed to check if the attributes were relevant.

**Table 2:** Example of choice set provided to OBs-GYNs in the discrete choice experiment

Attribute	Job A	Job B
Minimum duration of service	3 years	5 years
Opportunity of professional development	Available	Not available
Provision of education for children	Up to primary	Up to secondary
Availability of technology	No facility	Both
Team in job setting	OBGY and AA	OBGY, Paed,and Anesthetic
Monthly salary	Rs. 150 000	Rs. 200 000
Opportunity of Private practice	Available	Not available
Provision of Car allowance	Not available	Available
I will choice	Job A	Job B
	None of the above	

### Data collection

A total of 370 OBs-GYNs were working in Nepal at the time of the study. Among them,two were on maternity leave, 13 were out of the country, and 30 had participated in the pretesting. So, 325 OBs-GYNs were eligible for inclusion in the study. Upon approaching all 325 OBs-GYNs, 189 agreed to participate in the study (response rate, 58.15%). The study could not, however, collect the data on the background characteristics of those who did not participate in the survey.

Three enumerators, including the first author, carried out the data collection between January and March 2016. The participants completed a brief questionnaire with information on demographics, education, and work experiences. Then, an introductory script was read to the participants to acclimatize them to the hypothetical nature of the DCE. The introductory script directed participants to consider that the presented DCE job scenarios were all located in district hospitals. In this manner, the elicited responses reflected preferences for job attributes conditional on a district hospital setting. Following the introductory script, the participants proceeded to complete the survey questionnaire that comprised two hypothetical job scenarios. Participants were asked to select one among the available alternatives including “none of the above” in case both the options were felt unattractive.

### Ethical considerations

Ethical Review Board (ERB) at the Institute of Medicine, Tribhuvan University, approved the study protocol. The study objectives were explained before requesting the OBs-GYNs to participate in the study. Moreover, the right of participants to voluntary participation and withdrawal from the study at any stage was ascertained. To assure the confidentiality of the data provided, access to the dataset was granted only to the study team. Each participant provided written consent for participation in the study.

### Analysis

The data were entered into SPSS version 21 and used for further statistical analyses, which was performed in R 3.2.4. A descriptive analysis was carried out for the demographic characteristics of the participants. Multinomial logistic regression models were run using mlogit package in R to analyze the participants’ choice decision as a dependent variable. First, a simple multinomial logistic regression model was estimated for the job attributes. Then, for sub-group analysis, interactions of independent different variables were entered into the regression models. Marginal utility coefficients and standard errors were estimated to find out the relative importance placed on the job attributes. The willingness to pay/accept was calculated as the ratio of the coefficients of the attribute to the coefficients of the salary attribute.

## Results

Overall, 189 (58.15%) OBs-GYNs responded to the questionnaire. The background characteristics of the respondents are shown in Table 3. The statistics show that 64.6% of them were females and 86.2% were married. Only14.3% of the OBs-GYNs were aged greater than 45 years. Regarding education, 87.8% held an MD degree and 36.5%graduated from foreign universities. A majority (48.1%) worked in medical colleges, followed by private hospitals (22.2%) and government health institutions (21.7%).

**Table 3:** Descriptive characteristics of the participating OBs-GYNs

Characteristics	Frequency (%)
Gender	
Female	122 (64.56)
Male	67 (35.44)
Age	
< 35 years	81 (42.87)
36–45 years	81 (42.87)
> 45 years	27 (14.26)
Marital status	
Married	163 (86.24)
Unmarried	26 (13.76)
Religion	
Hindu	166 (87.83)
Buddhist	14 (7.41)
Muslim	8 (4.23)
Christian	1 (0.53)
Highest education attained	
MD	166 (87.83)
Diploma in Gynecology and Obstetrics	11 (5.82)
Others	12 (6.35)
Country of highest education	
Nepal	120 (63.49)
China	22 (11.64)
India	11 (5.82)
Russia	10 (5.29)
Bangladesh	8 (4.23)
Kyrgyzstan	6 (3.17)
Others	12 (6.36)
Type of workplace	
Government facility	41 (21.70)
Medical college	91 (48.15)
Private hospital	42 (22.22)
Others	*15 (7.93)*

The job preferences of OBs-GYNs in a district hospital are presented in Table 4. The study found statistically significant coefficients for the lower minimum duration of service, provision of primary and secondary education to children, presence of pediatrician and anesthesiologist with OB-GYN in the team at the workplace, opportunity of private practice, and provision of car allowance. As indicated by the sign and values of marginal utility, a full team (OB-GYN, pediatrician, and anesthesiologist) at the workplace had the highest marginal utility (0.4) and willingnesstopay NPR 195749 (USD 1673) per annum. It was followed by the provision of primary and secondary education for children (0.2, 0.3) and willingnesstoaccept (sacrifice) NPR 85646 (USD 732) and NPR 118592 (USD 1013) per annum, respectively. Similarly, the private practice opportunity received third preference order with the marginal utility of 0.1 and willingnesstopay NPR 52288 (USD 447) per annum. On the other hand, a higher minimum duration of service in the district has disutility (− 0.3) and willingnesstoaccept NPR 123602 (USD 1026) per annum for additional 1 year of minimum duration of service in the district hospitals.

**Table 4:** Results of the multinomial logit model

Attribute	Marginal utility	SE	WTA (NPR)
Main effects			
Constant	− 1.833***	0.218	
Minimum duration of service	− 0.262***	0.036	− 123 602
Monthly salary	2.1 × 10^−06^	1.1 × 10^−06^	
Opportunity of professional development	0.040	0.033	19 244
Provision of secondary education to children	0.252***	0.042	118 592
Provision of primary education to children	0.182***	0.045	85 646
Either telemedicine or video conferencing available at the workplace	− 0.055	0.041	− 25 963
Both telemedicine and video conferencing available at the workplace	− 0.079	0.044	− 37 300
Team of OB-GYN, pediatrician, and anesthesiologist at the workplace	0.416***	0.033	195 749
Opportunity of private practice	0.111***	0.032	52 288
Provision of car allowance	− 0.070*	0.031	− 33 226
Interaction effects			
Opportunity of private practice × gender (female)	− 0.130	0.069	
Team of OB-GYN, pediatrician, and anesthesiologist at the workplace × age (> 45 years)	− 0.235	0.147	
Team of OB-GYN, pediatrician, and anesthesiologist at the workplace × age (36–45 years)	− 0.262**	0.084	
Both telemedicine and video conferencing available at the workplace × age (> 45 years)	− 0.303*	0.132	
Both telemedicine and video conferencing available at the workplace × age (36–45 years)	− 0.139	0.083	
Team of OB-GYN, pediatrician, and anesthesiologist at the workplace × total work experience (5–10 years)	− 0.130	0.092	
Opportunity for private practice × highest education attained (extra degree)	− 0.414*	0.196	
Opportunity for private practice × highest education attained (MD)	− 0.033	0.148	
Team of OB-GYN, pediatrician, and anesthesiologist at the workplace × highest education attained (extra degree)	− 0.460*	0.209	
Team of OB-GYN, pediatrician, and anesthesiologist at the workplace × highest education attained (MD)	− 0.334*	0.159	
Both telemedicine and video conferencing available at the workplace × no experience of working in remote area	0.185	0.111	
Provision of secondary education to children × country of highest education (outside Nepal)	− 0.228**	0.084	
Provision of primary education to children × country of highest education (outside Nepal)	− 0.180*	0.086	
Opportunity for professional development × type of workplace (medical college)	0.207*	0.087	
Opportunity of professional development × type of workplace (private hospital)	0.153	0.097	
Monthly salary × type of workplace (medical college)	1.2 × 10^−6^	1.2 × 10^−6^	
Monthly salary × type of workplace (private hospital)	− 2.5 × 10^−6^*	1.2 × 10^−6^	

*p< 0.05, **p< 0.01, ***p< 0.001

The coefficients with interaction terms in the models revealed that the preference for the job attributes changes across certain background characteristics of the OBs-GYNs. For instance, in the model with the main effects, having a full team at the workplace (OB-GYN, pediatrician, and anesthesiologist) had a marginal utility of 0.4 which reversed to a disutility of 0.3 when the interaction effect with participant’s agegroup of 36–45 years was introduced into the model. Likewise, in the main effects model, having the opportunity of professional development had no significant marginal utility. However, the interaction effect with medical college as the workplace of the OBs-GYNs revealed that the opportunity of professional development had a utility of 0.2.

### Sub-group analysis

The results of DCE stratified by the sub-groups suggest that the value placed on attributes in district hospitals varies within the sub-groups (see Additional file 1). The willingnesstopay was the highest for the presence of a full team at workplace (OB-GYN, pediatrician, and anesthesiologist) followed by opportunity of private practice and provision of secondary education to children, while the order of preference for female respondents was a full team at workplace (OB-GYN, pediatrician, and anesthesiologist) followed by provision of secondary education and primary education to children (see Additional file 1: Table S1). This indicates that females put relatively more emphasis on the education of children.

Unmarried OBs-GYNs valued a full team at the workplace (OB-GYN, pediatrician, and anesthesiologist) and the provision of secondary education to children. On the other hand, married OBs-GYNs valued lower minimum duration of service, provision of primary and secondary education to children, a full team at the workplace (OB-GYN, pediatrician, and anesthesiologist), the opportunity of private practice, and a provision of car allowance (see Additional file 1: Table S2).

Though the value placed on individual attributes varied to some extent, the attributes that emerged to be of significance remained the same when the analysis was confined to OBs-GYNs with an MD degree as their highest education (see Additional file 1: Table S3).

When comparing the attributes preferred by the OBs-GYNs of different age groups, we observed that opportunity of professional development and the provision of car allowance were valued by young OBs-GYNs only. Interestingly, while a full team at the workplace (OB-GYN, pediatrician, and anesthesiologist) added utility for OBs-GYNs aged < 35 years and 35–45 years, it emerged as a disutility for OBs-GYNs aged > 45 years (see Additional file 1: Table S4).

## Discussion

Our study presents inferences from the interviews with 189 OBs-GYNs about preferences for various job attributes to work in district hospitals of Nepal. We hope the findings of the study will be helpful to the Government of Nepal to encourage the recruitment and retention of OBs-GYNs including medical doctors and specialists in these hospitals of the country. Similarly, the Government of Nepal is pursuing the SDGs, and WHO has suggested 4.5 health workers including a doctor per 1000 population [15]. In order to make progress in this direction, strong incentive schemes and rural deployment strategies are necessary. This study intends to fulfill the evidence gap in this regard. Similarly, though Nepal has achieved considerable progress in maternal health indicators over the decade [16], a further progress in availability and accessibility maternal health services is necessary to control all kinds of preventable maternal deaths. For this, the availability of OBs-GYNs in district hospitals is crucial to deliver quality maternal and child health services. As OBs-GYNs in the hospitals are well positioned for timely evaluation of the patient condition, proper diagnosis, and prompt treatment, they can bring about a significant improvement in maternal morbidity and mortality rates [2].

In order to materialize the national plans and program objectives, this study has come up with some interesting results. While the OBs-GYNs did not put a relatively higher value on some of the important job attributes like opportunities of personal professional development, the provision of primary and secondary education to the children appeared to be an important consideration while making rural job decision at district hospitals of Nepal. This pattern was consistent for all sub-group analyses except among OBs-GYNs aged > 45 years. Currently, under the Government of Nepal’s target of achieving a 100% primary school enrolment, initiatives such as establishing schools in remote areas and strengthening schools via placement of competent school teachers and adequate logistics are being carried out [17, 18]. Our findings suggest that these efforts, targeted at increasing the school enrolment rate, can be symbiotic to attracting OBs-GYNs to serve in district hospitals. However, options of private schools—which are considered superior in terms of the quality of education—are limited in the remote areas of Nepal [19]. Research in the future should focus on exploring if there are preference differentials for private and public school education for OBs-GYNs children.

OBs-GYNs’ preference for private practice was also reflected in the experiment. In Nepal, private practice at clinics other than public hospitals is common, and it is a major contribution to the doctor’s income [20]. Though the private clinics are comparatively expensive to receive health services, patients perceive these services to be readily accessible and reliable [21, 22]. In this setting, it seems plausible that the OBs-GYNs highly value the availability of private practice while deciding where to serve. In the local context, as discussed in the introduction, district hospitals are, in general, located in the district headquarter where private service providers are also available. On the other hand, the monthly salary of OBs-GYNs is very low in comparison with neighbor countries; the private practice opportunity allows for extra income. So, most OBs-GYNs preferred private practice beyond their official hours. Generally, district hospitals are located in the districts headquarter and is the market center for the population in the districts. In this scenario, the OBs-GYNs have the opportunity to practice privately in nearby outlets, private polyclinics, and private hospitals.

OBs-GYNs expressed preferences for the availability of a full team at the workplace (OB-GYN, pediatrician, and anesthesiologist). The good performance of an OB-GYN depends on the competency and support of pediatrician, anesthesiologist, nursing staff, radiologist, and other staf f[23]. It also underscores the point that for sustainable retention of OBs-GYNs in district hospitals, a holistic approach in recruiting a complete team of specialists is necessary. In the long run, in addition to recruiting OBs-GYNs, recruiting and strengthening other health cadres also need to be prioritized.

On the contrary, a higher mandatory duration of service in district hospitals was considered as a disincentive. It is possible that OBs-GYNs equate a higher mandatory duration of service in remote areas with limited access to opportunities for professional growth and training opportunities that are available in the capital city. Similar to our findings, in a DCE among medical students in Ghana, the students identified having to work for 2 years (vs. having to work for 5 years) before they could receive a study leave as attractive when selecting hypothetical job postings [24].

Findings from the willingness to pay/accept analyses quantified the financial incentive that would be required in return if the job attributes are present/absent in the package. Our finding on the attractive trait of financial incentives conforms to the global experiences. In Cameroon, medical and nursing students valued financial incentives in the form of “retentionbonus” as attractive in a DCE to build strategies for the retention in the health institutions [25]. Building on the evidence that financial incentives can attract doctors, the German government introduced legislation in 2011 to provide per patient financial incentives to the doctors serving in rural areas [26].

As the Government of Nepal targets to provide specialist care all over the country homogenously, the availability of sufficient OBs-GYNs is therefore crucial and at the same time can be a challenge. Although the crude number of OBs-GYNs trained in Nepal until 2012 is the highest when compared with other specialists [27], it is still far from sufficient if the specialized service is to be extended down to the district hospitals. On the positive note, the Government of Nepal has planned to establish medical colleges in each of the seven provinces in line with the recommendation of the High-Level Commission for Health Profession Education Polic y[28]. Similarly, the National Health Sector Strategy (NHSS) (2015–2020) has committed to move towards Universal Health Coverage (UHC) and ensure quality health services at all levels of the health system. The findings of this study in recruitment and retaining the human resource for health in CEONC center could help in devising strategies to fulfill the vacant post of OBs-GYNs in the CEONC centers to provide quality service and increase the number of OBs-GYNs in government sectors [29].

We acknowledge that our study has a few limitations. First, the stated preference method used, even if it allowed us to determine the relative strengths of the different attributes explored, cannot fully anticipate the decisions eventually made by participants in real-life situations. Only following them over a period of time, we will be in a position to ascertain that the job choices are actually made and in specific circumstances. Second, we did not collect information from the non-respondents and thus were unable to assess whether selection bias may have been incurred from differential participation; therefore, the results reflect the preferences of OBs-GYNs who participated in the survey and thus cannot generalized for those who denied participation in the study. Third, the study presented only for district hospital job options; the results cannot be used to model the uptake of rural vs. urban postings. Lastly, as the results are based on a DCE, the results need to be validated with the reported preference data, preferably from policy experiments.

## Conclusions

Our findings present the important job attributes that should be considered during the recruitment and retention of OBs-GYNs in the district hospitals of the Nepalese health system. While the presence of a team of OB-GYN, pediatrician, and anesthesiologist at the workplace; provision of primary and secondary education for children; and opportunity of private practice attracted OBs-GYNs, on the other hand, a higher minimum duration of service in district hospitals and the provision of a car allowance were taken as a disincentive. This study confirmed the importance of the combination of non-monetary interventions in attracting and retaining health workers in district hospitals in Nepal. Money is not the only factor that affects preferences to take up a rural post. Addressing OBs-GYNs’ job preferences strategically would, therefore, help the nation solve the recruitment and retention issues particularly at the district hospitals of the country.

## Supplementary information

**Additional file 1:** Table S1-Table S3.

## Data Availability

Data and materials are available from the corresponding author upon request.
